# Chicago Academy of Medical Science

**Published:** 1861-03

**Authors:** E. L. Holmes

**Affiliations:** Secretary; Chicago


### CHICAGO ACADEMY OF MEDICAL SCIENCE.

The regular monthly meeting of the Academy was held at its rooms this evening. The President Dr. Bloodgood in the chair. After the reading of the minutes of the last meeting, the President appointed the following committees in accordance with Art. VI. Sec. I. (revised,) of the constitution:

*Anatomy and Physiology*, Dr E. Powell,
*Surgery*, Prof. J. W. Freer, M. D.
*Theory and Practice*, Dr. A. Fisher,
*Obstetrics, and Diseases of Women*, Dr. O. Smith,
*Chemistry and Meteria Medica*, Dr J. H. Rauch,
*Public Health*, Prof. N. S. Davis, M. D.
*Pathology and Histology*, Prof. J. H. Hollister, M. D.

Dr. R. C. Hamil read an interesting report of a case of inversio uteri. The labor was conducted with comparatively little difficulty, although there was a slight antero-posterior contraction of the pelvis. For a couple of hours before the birth of the child, five or six drops of chloroform were administered from time to time on account of nervous excitement. Ten or fifteen minutes after the expulsion of the child, a violent pain seized the patient, which was immediately followed by the delivery of the placenta. An alarming loss of blood with great prostration ensued. On examination, the uterus was found inverted. Fortunately, after some efforts the organ was replaced; although there were subsequent symptoms of metritis, they were relieved by quinine, Veratrum Viride and Hyoscyamus, and the patient soon recovered perfect health. It should be observed that no force was applied to the cord in the delivery of the placenta.

Dr. H. N. Hurlbut reported orally a case in which he was recently called to a patient, married, aged about 40 years, suffering from violent hemorrhage and prostration. On examination he found the uterus inverted and presenting evident post-partum appearances. He reduced the organ readily, and the patient recovered without unfavorable symptoms. No child or placenta was any where observed, and no satisfactory account of the case could be obtained from the family.

Dr. Bevan related the following case to show the length of time which the lifeless fœtus may remain in the uterus. The patient aged 35, in the fifth or sixth month of pregnancy, stated that the motions of the child had suddenly ceased in consequence of injury received in a quarrel followed by violent exercise. No fœtal pulsations could be heard, and no motions could be induced either by pressure or by the application of cold to the abdomen. In three weeks the woman was delivered of a fœtus in a state of decomposition. No blood passed from the uterus and vagina except in the form of dark decomposed clots.

The subject of Pneumonia, assigned at the last meeting, was discussed at some length by Drs. O. Smith, Johnson, Heydock, Fisher, Davis, Wickersham, Bevan, Hamil, Andrews, McAllister and Bloodgood.

Subject for discussion at the next meeting: The diagnosis of cerebral diseases of children. Adjourned.

E. L. HOLMES, *Secretary.*

Chicago, February 1st, 1861.

